# Impact of multikinase inhibitors in reshaping the treatment of advanced gastroenteropancreatic neuroendocrine tumors

**DOI:** 10.1530/ERC-25-0052

**Published:** 2025-06-18

**Authors:** Alexander R Siebenhüner, Julie Refardt, Guillaume P Nicolas, Reto Kaderli, Martin A Walter, Aurel Perren, Emanuel Christ

**Affiliations:** ^1^ Department of Gastroenterology and Hepatology, University Hospital Zurich, and University of Zurich, Zurich, Switzerland; ^2^ Department Hematology and Oncology, Hirslanden Klinik Zurich, Zurich, Switzerland; ^3^ Department of Endocrinology, Diabetes, and Metabolism, Basel University Hospital, Center of Endocrine and Neuroendocrine Tumors, University of Basel, Basel, Switzerland; ^4^ Department of Internal Medicine, Section Endocrinology, Erasmus Medical Center Rotterdam, Rotterdam, The Netherlands; ^5^ Clinic for Radiology and Nuclear Medicine, Center for Neuroendocrine and Endocrine Tumors, University Hospital Basel, Basel, Switzerland; ^6^ ENETS Center of Excellence, Basel, Switzerland; ^7^ Department of Visceral Surgery and Medicine, Bern University Hospital, University of Bern, Bern, Switzerland; ^8^ Department of Nuclear Medicine, St. Anna Hospital, University of Lucerne, Lucerne, Switzerland; ^9^ Institute of Tissue Medicine and Pathology, University of Bern, Bern, Switzerland

**Keywords:** neuroendocrine tumor (NET), targeted therapy, everolimus, sunitinib, peptide receptor radionuclide therapy (PRRT), survival, predictive biomarker, treatment sequence

## Abstract

Neuroendocrine tumors (NETs) pose a considerable challenge due to their increasing incidence and frequently late-stage diagnosis. The arrival of multikinase inhibitors (MKIs) into clinical practice has brought notable progress in the management of advanced gastroenteropancreatic neuroendocrine tumors (GEP-NETs). This review aims at exploring the impact of MKIs in reshaping the treatment landscape for advanced GEP-NETs. Current approaches in managing advanced GEP-NETs are discussed, including somatostatin analogs, surgery, peptide receptor radionuclide therapy, and approved systemic treatments such as everolimus or sunitinib. The limitations and challenges faced in treating these tumors remain significant. Here, we review the clinical evidence supporting the use of everolimus as a targeted therapy, which has demonstrated improved progression-free survival (PFS), and the need for alternative therapies. Discussions focus on the clinical effectiveness and the emerging role of both established and novel MKIs in the treatment of GEP-NETs, including recent evidence from the CABINET trial and other emerging agents such as surufatinib, axitinib, pazopanib, and lenvatinib. We explore the clinical evidence that showcases sunitinib’s and other MKIs’ effectiveness in prolonging PFS compared to placebo in advanced GEP-NETs. Recently, MKIs have shown to have a significant impact for the treatment of advanced GEP-NETs. There remain several unmet needs that must be addressed, particularly regarding optimal treatment sequencing and the development of predictive biomarkers. Ongoing research and the use of current and emerging MKIs hold great potential to advance the treatment landscape for advanced GEP-NETs significantly.

## Introduction

Neuroendocrine tumors (NETs) represent a heterogeneous group of neoplasms that primarily affect the gastrointestinal (GI) tract, the pancreas and the lungs (Siegel *et al.* 2019). The incidence rate of NETs is relatively low, with only 1–5 cases per 100,000 people (Das & Dasari 2021). Nevertheless, over the past decade, there has been a notable global rise in NET cases, mainly due to the increased detection of early-stage tumors (Siegel *et al.* 2019, Yao *et al.* 2008*a*) and demographic changes. Most NETs are low- to intermediate-grade (G1 or G2) tumors, which are by definition well-differentiated and often present with mild or no symptoms unless the tumor secretes hormones or metabolites. Secreting NETs account for approximately 25% of all NETs (Halperin *et al.* 2017). Early NETs are increasingly detected in sites that are examined endoscopically, such as rectum and stomach only. As a result, many NETs remain undetected during their early stages. In contrast, 40–50% of NETs are diagnosed as locally advanced or metastatic tumors, making them unsuitable for radical curative-intent surgery (Strosberg *et al.* 2017). The treatment approach for locally advanced or metastatic NETs focuses on controlling tumor growth and managing functional symptoms such as flush and diarrhea, and local symptoms due to metastasis (Shah *et al.* 2018).

A hallmark of NET is the widespread overexpression of somatostatin receptors, particularly the subtype 2 (SSTR-2), on the surface of 80–90% of tumors. These receptors play a crucial role in both diagnosing and treating NET. The primary approach for managing symptoms and improving progression-free survival (PFS) in SSTR-2-positive mainly well-differentiated (G1, G2) NET involves somatostatin analogs (SSA) as the first-line treatment option. In cases of NET progression, different treatment modalities can be employed, although the specific sequences are not generally established and depend on individual patient characteristics and the availability of treatments (Strosberg *et al.* 2011).

Histopathological analysis of NETs reveals characteristics that underlie their response to targeted therapies. The heterogeneity in their molecular profile, including variations in growth factor receptor expression and downstream signaling pathways activation, directly influences their susceptibility to therapeutic agents such as everolimus and MKIs. Understanding these histopathological features is crucial for predicting treatment response and developing personalized therapeutic approaches (Barbieri *et al.* 2023).

The mammalian target of rapamycin (mTOR) is a main driver pathway in most NETs. This key protein is implicated in tumor growth, angiogenesis, and proliferation and has been previously described in the pathogenesis of GI-NETs (Kasajima *et al.* 2011). In addition, NETs commonly overexpress tyrosine kinases and their receptors, specifically receptors for the vascular endothelial growth factor (VEGF) and the platelet-derived growth factor (PDGF). These overexpressed tyrosine kinase receptors have been exploited as therapeutic targets to block tumor progression and dissemination in advanced tumor settings (Casanovas *et al.* 2005). In particular, pancreatic NETs (PanNETs) display high expression of platelet-derived growth factor receptors (PDGFR) α and β and the stem cell factor receptor (c-KIT), making PanNETs susceptible to systemic treatment strategies with tyrosine kinase inhibitors (TKIs) and multikinase inhibitors (MKIs). MKIs can inhibit various tyrosine kinases simultaneously, thereby inhibiting signaling pathways involved in tumor growth and angiogenesis (Zhang *et al.* 2013, Carmona-Bayonas *et al.* 2017). It is important to note that the susceptibility of PanNETs to TKIs and MKIs may vary among individual tumors, and the effectiveness of these systemic therapies may depend on various factors such as tumor grade, tumor stage, and specific molecular characteristics. In some cases, combining TKIs or MKIs with other treatment modalities, such as peptide receptor radionuclide therapy (PRRT), may enhance the therapeutic response and improve outcomes for patients with PanNETs.

This review explores the benefits of TKIs and MKIs and their impact on the management of advanced gastroenteropancreatic (GEP-NETs), with particular attention to both established agents and emerging therapeutic options that are reshaping the treatment landscape.

## Preclinical evidence supporting MKIs in GEP-NETs

Before discussing clinical applications, it is important to understand the preclinical rationale supporting MKI use in NETs. Several *in vitro* and *in vivo* studies have established the scientific basis for multiple kinases therapy in NETs.

Cabozantinib has demonstrated significant preclinical activity in NET models. Studies by Bentzien *et al.* (2013) showed that cabozantinib effectively inhibits MET and VEGFR2 phosphorylation, leading to decreased cell migration, invasion, and angiogenesis in NET cell lines. In addition, research by Cives *et al.* (2020) revealed that cabozantinib disrupts the tumor microenvironment by targeting tumor-associated macrophages and fibroblasts, reducing their pro-tumorigenic activities in NET models. These studies provided the rationale for subsequent clinical investigations.

For lenvatinib, preclinical investigations have shown potent anti-angiogenic and anti-tumor effects across multiple NET subtypes. Di Mauro *et al.* (2022) demonstrated that lenvatinib inhibits VEGFR1–3, FGFR1–4, PDGFRα, RET, and KIT, effectively suppressing tumor growth and angiogenesis in xenograft models of NETs. The drug’s ability to target multiple kinases simultaneously appears particularly advantageous in overcoming potential resistance mechanisms.

Surufatinib’s unique mechanism, targeting VEGFR, FGFR1, and CSF-1R, has been extensively studied by Wu *et al.* (2021). Their research showed that surufatinib not only inhibits angiogenesis but also modulates the immune microenvironment by affecting tumor-associated macrophages through CSF-1R inhibition. This dual action provides a strong preclinical rationale for its use in NET treatment.

These preclinical studies collectively provide strong evidence for the efficacy of MKIs in NET treatment and have informed the design of subsequent clinical trials exploring their therapeutic potential.

## Current treatment landscape for managing advanced GEP-NETs

The management of advanced GEP-NETs has evolved considerably in recent years, with a growing emphasis on personalized treatment approaches based on (molecular) tumor characteristics, patient factors, and biomarker profiles. As discussed by Fazio & La Salvia (2023), precision medicine in GEP-NETs requires integration of multiple factors including primary tumor site, grade, functional status, somatostatin receptor expression, and molecular profile to guide treatment decisions. This comprehensive approach has led to more tailored treatment strategies and improved outcomes for patients with advanced disease.

**Figure 1:**
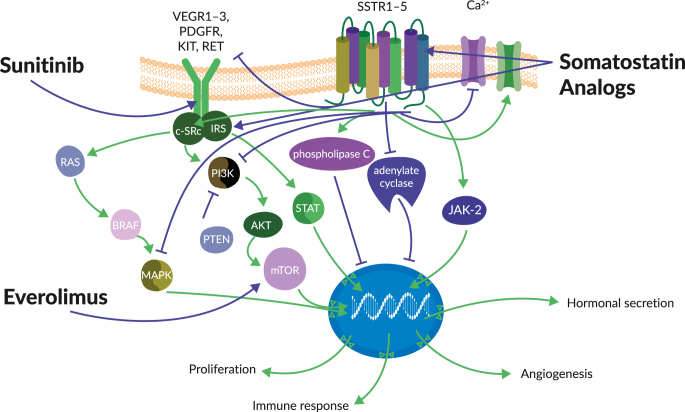
Potential molecular targets in NET and SSA crosstalk of sunitinib and everolimus. Adapted from Carmona-Bayonas *et al*. (2017). VEGFR, vascular endothelial growth factor; PDGFR, platelet-derived growth factor receptor; SSTR, somatostatin receptor; SSA, somatostatin analogs; MAPK, mitogen-activated protein kinase; Akt, protein kinase B (PKB), also known as Akt; mTOR, mammalian target of rapamycin; PTEN, phosphatase and tensin homolog; JAK-2, Janus kinase 2.

The treatment options for advanced low- to intermediate-grade (G1/G2) tumors range from surgery and local ablative methods to somatostatin analogs and PRRT or, for certain types of low-grade NETs, targeted drugs such as everolimus or sunitinib may be used to block specific pathways such as mTOR or VEGF (Fig. 1). Everolimus is classified as an mTOR inhibitor and inhibits downstream signaling by binding to FKBP-12 (Yao *et al.* 2008*b*). In contrast, sunitinib acts by inhibiting multiple tyrosine kinases, including VEGFRs, PDGFRs, and KIT. Pivotal phase 3 clinical trials for everolimus and sunitinib are summarized in Table 1.

**Table 1:** Summary of pivotal phase 3 clinical trial evidence for everolimus and sunitinib in advanced GEP-NETs.

Study (NCT)	Study type	NET primary origin	Tumor grade	No. patients	Treatment vs placebo (*n*)	MKI (therapy line)	Primary endpoint	Key outcomes	Prior surgery, %	Prior PRRT, %	Biomarkers	Ref
**Everolimus (mTOR inhibition)**												
RADIANT-2 (NCT00412061)	Phase 3, double-blind, placebo-controlled multicenter study	NET (∼50% small intestine)	G1–G2	429	Everolimus plus octreotide LAR (*n* = 211) vs placebo plus octreotide LAR (*n* = 204)	>1	PFS	Median PFS was 16.4 (95% CI, 13.7–21.2) vs 11.3 (95% CI, 8.4–14.6) months (HR 0.77, 95% CI 0.59–1.00)	NR	NR	CgA	Pavel *et al.* (2011)
	Open-label extension crossover study of RADIANT-2	NET (∼50% small intestine)	G1–G2	170	Open-label everolimus (*n* = 143 crossover from placebo; *n* = 27 continued on everolimus)	>1	PFS; final OS reported in open-label analysis	OS was 29.2 (95% CI, 23.8–35.9) months for everolimus vs 35.2 (95% CI, 30.0–44.7) months for placebo	n/a	n/a	n/a	Pavel *et al.* (2017*a*), Pavel *et al.* (2019)
RADIANT-3 (NCT00510068)	Phase 3, double-blind, placebo-controlled multicenter study	PanNET	G1–G2	410	Everolimus (*n* = 207) vs placebo (*n* = 203) plus best supportive care	>1	PFS	PFS was 11 vs 4.6 months (HR 0.35, 95% CI 0.27–0.45; *P* < 0.001)	NR	Within 4 weeks before randomization were excluded	CgA, NSE, PIGF, sVEGF1&2, VEGF-A, bFGF	Yao *et al.* (2011)
	Open-label extension study of RADIANT-3	PanNET	G1–G2	225	Open-label everolimus (*n* = 172 crossover from placebo; *n* = 53 continued on everolimus)	>1	Final OS analysis	Median OS was 44.0 months (95% CI, 35.6–51.8 months) for those who continued on everolimus and 37.7 months (95% CI 29.1–45.8 months) for those who crossed over from placebo (HR 0.94, 95% CI, 0.73–1.20; *P* = 0.30)	n/a	n/a	n/a	Yao *et al.* (2016*b*)
RADIANT-4 (NCT01524783)	Phase 3, double-blind, placebo-controlled, multicenter randomized study	GI or lung NET	G1–G2	302	Everolimus (*n* = 205) vs placebo (*n* = 97)	>1	Primary endpoint was PFS	PFS was 11 (95% CI, 9.2–13.3) months in the everolimus arm vs 3.9 (955 CI, 3.6–7.4) months in the placebo arm; everolimus was associated with a 52% reduction in the estimated risk of progression or death (HR, 0.48; 95% CI, 0.35–0.67; *P* < 0.00001); OS not reached	59 vs 72% (everolimus vs placebo arms)	22 vs 20% (everolimus vs placebo arms)	CgA, NSE	Yao *et al.* (2016*a*)
**Sunitinib (tyrosine kinase inhibition)**												
SU-1111 (NCT00428597)	Phase 3, double-blind, placebo-controlled, multicenter, randomized study	PanNET	G1–G2	171	Sunitinib (*n* = 86) vs placebo (*n* = 85)	>1	Primary endpoint was PFS	PFS was 11.4 vs 5.5 months (HR for progression or death 0.42, 95% CI 0.26–0.66; *P* < 0.001); OS not reached; no crossover – early terminated due to survival benefit of sunitinib	88 vs 91% (sunitinib vs placebo arms)	10 vs 14% (sunitinib vs placebo arms)	NR	Raymond *et al.* (2011)
	Open-label extension crossover study	PanNET	G1–G2	171 (160 with completed scan sites/timepoints)	Sunitinib (*n* = 86) vs placebo (*n* = 85)	>1	Final median OS	PFS was 12.6 (95% CI 11.1–20.6) months for sunitinib and 5.8 (95% CI 3.8–7.2) months for placebo (HR, 0.32; 95% CI 0.18–0.55; *P* = 0.000015). 5 years after study closure, median OS was 38.6 (95% CI 25.6–56.4) months for sunitinib and 29.1 (95% CI 16.4–36.8) months for placebo (HR 0.73, 95% CI 0.50–1.06; *P* = 0.094), with 69% of placebo patients having crossed over to sunitinib	n/a	n/a	n/a	Faivre *et al.* (2017)
	Post-hoc analysis of individual patient data from the pivotal phase 2 (NCT00056693) and phase 3 study (NCT00428597)	PanNET	G1–G2	237	Sunitinib (*n* = 152) vs placebo (*n* = 85)		Optimal RECIST (v.1.0) response cut-off value and most informative timepoint (highest AUC)	Reduction of 10% (vs baseline) achieved the highest sensitivity (50%) and specificity (82%) among the cut-offs tested. Month 7 was the most informative timepoint (AUC 0.78, 95% CI 0.66–0.9); odds ratio 1.05 (95% CI 1.01–1.08; *P* = 0.002)	n/a	n/a	n/a	Lamarca *et al.* (2018)

AUC, area under curve; bFGF, basic fibroblast growth factor; CI, confidence interval; CgA, chromogranin A; HR, hazard ratio; GET-NET, gastroenteropancreatic neuroendocrine tumor; GI, gastrointestinal; LAR, long-acting release; MKI, multikinase inhibitor; n/a, not applicable; NET, neuroendocrine tumor; NR, not reported; NSE, neuron-specific enolase; OS, overall survival; PLGF, placental growth factor; PanNET, pancreatic NET; PFS, progression-free survival; PRRT, peptide receptor radionuclide therapy; RECIST, Response Evaluation Criteria in Solid Tumors; VEGF, vascular endothelial growth factor; sVEGF 1/2, soluble VEGF 1/2.

For patients with pancreatic NETs (PanNETs), chemotherapy remains an important treatment option, particularly streptozotocin-based regimens. Recent research by Fanciulli *et al.* (2023) identified potential predictive factors for response to streptozotocin in PanNETs, including Ki-67 index, tumor burden, and prior treatments. This highlights the importance of patient selection and the need for biomarkers to guide chemotherapy use in the era of targeted therapies and PRRT.

On the other hand, well-differentiated, high-grade (G3) tumors, which are classified as G3 NET, are typically treated more frequently with chemotherapy (Basturk *et al.* 2015, Pavel *et al.* 2016). The role of PRRT in this setting is still under investigation, and treatment decisions should be individualized based on tumor characteristics and patient factors.

## mTOR inhibition with everolimus in GEP-NETs

Pivotal, double-blind, placebo-controlled, multicenter phase 3 studies evaluating everolimus include RADIANT-2 (Pavel *et al.* 2011), RADIANT-3 (Yao *et al.* 2011) and RADIANT-4 (Yao *et al.* 2016*a*). These trials have demonstrated mTOR inhibition activity in a range of advanced NETs (Table 1).

In the double-blind, randomized-controlled trial and the open-label extension of RADIANT-2, combining the mTOR inhibitor everolimus and the SSA octreotide long-acting release (LAR) in patients with various pretreated NETs, showed promising results in terms of tumor growth regression and PFS (Pavel *et al.* 2011, 2017*a*, 2019). In the primary analysis, the median PFS (primary endpoint) was 16.4 months in the everolimus and octreotide LAR group and 11.3 months in the placebo and octreotide LAR group (one-sided log-rank test; *P* = 0.026), with the combination resulting in a 23% risk reduction (hazard ratio (HR) 0.77; *P* = 0.026) of progression or death (Pavel *et al.* 2011). A post-hoc analysis of RADIANT-2 aimed to analyze the effect of everolimus on the pharmacokinetics (PKs) of octreotide LAR in patients with advanced NETs. The researchers evaluated the PK findings using data from 182 patients, with at least one evaluable blood everolimus and plasma octreotide (before the injection) concentration (Cmin). Although the co-administration of octreotide LAR to everolimus did not affect efficacy, the PK analysis revealed that an increased everolimus minimum concentration was associated with a higher risk for pulmonary and metabolic side effects (Pavel *et al.* 2017*b*).

The landmark RADIANT-3 trial in the field of advanced low- to intermediate-grade (G1/G2) NETs demonstrated increased PFS with everolimus (median PFS 11.0 vs 4.6 months; HR 0.35; 95% confidence interval (CI) 0.27–0.45; *P* < 0.001) (Yao *et al.* 2011). A sub-analysis of RADIANT-3 showed that previous chemotherapy did not affect the efficacy and toxicity of everolimus treatment (Lombard-Bohas *et al.* 2015). However, criticism of this trial mostly included a placebo-controlled design and the fact that everolimus did not demonstrate an overall survival (OS) advantage. Correcting for crossover in case of progression did also not significantly affect OS (Yao *et al.* 2016*b*).

The phase 3 RADIANT-4 trial emphasized the effectiveness of everolimus in treating well-differentiated (G1/G2) NETs of the lung and gastrointestinal tract (GI-NETs) (Yao *et al.* 2016*a*). This study aimed to provide a clearer understanding of the treatment outcomes by minimizing any confounding factors. It specifically focused on patients who had previously experienced progression after SSAs. Similar to results from RADIANT-2 and RADIANT-3 trials, placebo-controlled PFS in the everolimus treatment arm was significantly improved by about 7 months, with a 52% reduction in the estimated risk of progression with everolimus (HR 0.48; 95% CI 0.35–0.67; *P* < 0.00001) (Yao *et al.* 2016*a*). The objective findings of this study indicated that the observed effects can be attributed to mTOR inhibition. This conclusion is supported by the fact that no crossover or combination of SSAs was allowed in the study’s design, and patients were stratified based on prognostic factors such as the World Health Organization (WHO) performance status, tumor primary site and prior exposure to SSAs (Yao *et al.* 2016*a*).

The landmark RADIANT-4 trial (Yao *et al.* 2016*a*) resulted in FDA (U.S. Food and Drug Administration; https://www.fda.gov/) and EMA (European Medicines Agency; https://www.ema.europa.eu/) approvals of everolimus for the treatment of progressive, well-differentiated, non-functional, unresectable, locally advanced, or metastatic GI and lung NETs. The current guidelines from the European Neuroendocrine Tumor Society (ENETS), the North American Neuroendocrine Tumor Society (NANETS), the National Comprehensive Cancer Network (NCCN), and the European Society of Medical Oncology (ESMO) recommend everolimus for the treatment of progressive pancreatic NETs and G1/G2 non-functional NETs of GI or lung origin (Anthony *et al.* 2010, Boudreaux *et al.* 2010, Phan *et al.* 2010, Pavel *et al.* 2017*c*, 2020, Shah *et al.* 2018).

Although the combination of everolimus with SSA is not currently recommended in the ENETS or ESMO guidelines (Pavel *et al.* 2016, 2020), data exist that this combination might have synergistic effects. The combination of everolimus plus SSA was investigated in patients with functional NETs (mainly of small intestine origin) in the phase 3 RADIANT-2 trial, showing tumor size reduction, disease stabilization, and significant decreases in biomarker levels. However, the trial did not improve the primary endpoint PFS compared to octreotide in a central review (Pavel *et al.* 2011), and no significant difference in OS was observed for the combination even after adjusting for imbalances in the baseline covariates (Pavel *et al.* 2017*a*).

Antitumor effects in patients with PanNETs using combined mTOR and VEGF pathway–targeted therapy have also been evaluated in a two-stage, single-arm phase 2 trial (Hobday *et al.* 2015). In this trial, a promising response rate (RR) with 41%, resulting at a median PFS of 13.2 months and an improvement by a median OS of 34 months keeps an outlook for such combinations.

Taken together, mTOR inhibition with everolimus appears to be a valuable treatment option, increasing clinical responses and improving PFS rates in well-to-moderately differentiated NET. However, continued evaluation of mTOR combination studies is warranted (Hobday *et al.* 2015).

## Clinical evidence for sunitinib in GEP-NETs

Sunitinib, an oral TKI that targets multiple receptors, including VEGFR1‒3, PDGFRβ, c-KIT, FLT-3 and RET, has demonstrated antitumor activity in advanced PanNETs (Kulke *et al.* 2008). The phase 3 SU-1111 trial was pivotal in assessing the effectiveness and safety of sunitinib in this setting (Raymond *et al.* 2011). This double-blind, placebo-controlled, multicenter trial demonstrated that sunitinib significantly improved PFS compared to placebo. In advanced, well-differentiated PanNETs, the PFS was extended by ca 6 months compared to placebo, similar as everolimus (REF) (Raymond *et al.* 2011). Based on the findings from the SU-1111 trial, sunitinib was approved by the FDA and EMA for the treatment of progressing, unresectable, locally advanced, or metastatic, and well-differentiated PanNETs.

Regarding safety, the SU-1111 trial reported that 95% of patients in the sunitinib group experienced adverse events, with the most common being diarrhea (59%), nausea (45%), asthenia (34%), and vomiting (34%). Grade 3 or 4 adverse events occurred in 33% of patients receiving sunitinib, with the most frequent being neutropenia (12%) and hypertension (10%). Despite these adverse events, the safety profile was considered manageable with dose modifications and supportive care (Raymond *et al.* 2011).

Long-term follow-up data of SU-1111 showed an impressive doubling of PFS from 5.8 months with placebo to 12.6 months with sunitinib, which translated into a median OS improvement of nearly 10 months 5 years after study closure (Faivre *et al.* 2017). Furthermore, in a post-hoc analysis of previous phase 2 and 3 studies involving 152 patients treated with sunitinib (Lamarca *et al.* 2018), univariate analysis revealed a 10–30% reduction in the size of marker lesions on imaging as a significant predictor for improved PFS. In contrast, only the threshold of 10% remained statistically significant after multivariate analysis was applied. This result supports the survival benefit of sunitinib in patients with PanNETs (Lamarca *et al.* 2018).

## Emerging MKIs/TKIs in GEP-NETs

In addition to the approved MKI sunitinib, several phase 2/3 trials of newer MKIs demonstrated promising clinical activity in NETs. Surufatinib, a novel multi-target kinase inhibitor, selectively inhibits VEGFR1–3, FGFR1, and CSF-1R. Its unique targeting profile, particularly the inhibition of CSF-1R which regulates tumor-associated macrophages, provides potential advantages in the NET treatment landscape. This agent has shown remarkable effectiveness in both pancreatic and extrapancreatic NETs, with successful applications also being reported in advanced solid tumors.

The SANET trial program, consisting of trials for extrapancreatic NET (SANET-ep) and PanNET (SANET-p), demonstrated the effectiveness of surufatinib in phase 3 randomized trials (Xu *et al.* 2020*a*,*b*). For SANET-p, surufatinib significantly improved the median PFS (10.9 vs 3.7 months), while in the SANET-ep population, the improvement was similarly impressive (9.2 months (95% CI 7.4–11.1) vs 3.8 months for placebo). These trials suggest that surufatinib might be a valuable new TKI option after the prior failure of at least two systemic treatment lines in both PanNET and extrapancreatic NET settings.

Recent long-term follow-up data from the SANET-ep trial further confirmed surufatinib’s efficacy, showing sustained PFS benefits and a manageable safety profile with longer treatment duration. The most common adverse events included hypertension (36%), proteinuria (30%), and diarrhea (29%), with grade 3 or higher adverse events occurring in 66% of patients in the surufatinib group compared to 33% in the placebo group (Xu *et al.* 2025). Despite these toxicities, only 10.6% of patients discontinued treatment due to adverse events, suggesting acceptable tolerability with appropriate management.

Axitinib, a potent and selective second-generation inhibitor of VEGFR1, 2 and 3, has shown promise in NET treatment (Strosberg *et al.* 2016). Its high specificity for VEGF receptors potentially offers a more focused anti-angiogenic approach compared to broader spectrum MKIs. However, a phase 2 study evaluating axitinib in patients with extrapancreatic NETs reported relatively high rates of adverse events, particularly grade 3/4 hypertension, which may limit its use in unselected patients (Strosberg *et al.* 2016).

In this phase II study of axitinib, common adverse events included fatigue (76%), diarrhea (63%), and hypertension (60%). Grade 3/4 adverse events occurred in 63% of patients, with hypertension (24%), fatigue (16%), and diarrhea (16%) being the most frequent. The high rate of adverse events led to dose reductions in 70% of patients and treatment discontinuation in 23%, highlighting the importance of toxicity management when using this agent (Strosberg *et al.* 2016).

Pazopanib, which targets VEGFR1, 2, 3, PDGFR, and c-KIT, has demonstrated particular activity in PanNETs (Phan *et al.* 2015). A multicenter, single-group phase 2 study evaluating pazopanib in combination with depot octreotide for patients with well-differentiated NETs showed promising results, particularly in terms of PFS for PanNETs, although results were less impressive for small intestine NETs (Phan *et al.* 2015).

In the pazopanib study, all patients experienced at least one adverse event, with 77% experiencing grade 3 or higher toxicities. The most common grade 3/4 adverse events were hypertension (32%), neutropenia (25%), and fatigue (11%). Dose reductions were required in 17 patients (33%), and 13 patients (25%) discontinued treatment due to adverse events. These findings underscore the need for close monitoring and proactive management of toxicities when using pazopanib (Phan *et al.* 2015).

The phase 2 TALENT trial (GETNE1509) investigated lenvatinib, a multikinase inhibitor targeting VEGFR1–3, FGFR1–4, PDGFRα, RET, and KIT, after the failure of mTOR inhibitors or TKI in advanced G1/G2 PanNET or after SSA failure in GI-NETs (Capdevila *et al.* 2021). For midgut NETs, lenvatinib demonstrated encouraging results, with a radiological RR of 16.4%, a disease control rate of 92.7%, and a median PFS of 15.7 months (95% CI 12.1–19.5) (Capdevila *et al.* 2021).

A Japanese phase II study of lenvatinib in advanced neuroendocrine neoplasms also showed promising results, with a RR of 25.0% and a median PFS of 7.0 months. However, toxicity was significant, with 15.6% of patients discontinuing treatment due to adverse events. The most common grade 3 or higher adverse events included hypertension (50.0%), proteinuria (12.5%), and thrombocytopenia (9.4%) (Capdevila *et al.* 2021).

The recent phase III CABINET trial has further expanded the landscape of MKI therapy in NETs (Chan *et al.* 2025). This study investigated cabozantinib in patients with advanced well-differentiated grade 1/2 carcinoid tumors, demonstrating significant improvement in PFS compared to placebo. The study showed that cabozantinib achieved a median PFS of 11.4 months compared to 5.3 months with placebo (HR: 0.46, *P* < 0.001), establishing it as another potential treatment option for patients with progressive carcinoid tumors (Chan *et al.* 2025). A comparison of the recent MKI data is listed in Table 2.

**Table 2:** Summary of recent GEP-NET studies using different MKIs.

Study (NCT)	Study type	NET primary origin	Tumor grade	No. patients	Treatment vs placebo (*n*)	Primary endpoint	Key outcomes	Prior therapy	Biomarkers	Ref
SANET-p (NCT02589821)	Phase 3, double-blind, placebo-controlled	PanNET	G1–G2	172	Surufatinib (*n* = 113) vs placebo (*n* = 59)	PFS	Median PFS 10.9 vs 3.7 months (HR 0.49; *P* < 0.001)	Prior systemic treatments allowed	Not reported	Xu *et al.* (2020*a*)
SANET-ep (NCT02588170)	Phase 3, double-blind, placebo-controlled	Extrapancreatic NET	G1–G2	198	Surufatinib (*n* = 113) vs placebo (*n* = 59)	PFS	Median PFS 9.2 vs 3.8 months (HR 0.33; *P* < 0.001)	Prior systemic treatments allowed	Not reported	Xu *et al.* (2020*b*)
TALENT (GETNE1509) (NCT02678780)	Phase 2, single-arm, multicenter	PanNET and GI-NET	G1–G2	111	Lenvatinib (*n* = 111)	ORR, PFS	PanNET cohort: ORR 15.3%, mPFS 15.7 months; GI-NET cohort: ORR 16.4%, mPFS 15.7 months	Post-SSA failure	CgA	Capdevila *et al.* (2021)
CABINET (NCT03375320)	Phase 3, double-blind, placebo-controlled	Carcinoid tumors	G1–G2	197	Cabozantinib (*n* = 98) vs placebo (*n* = 99)	PFS	Median PFS 11.4 vs 5.3 months (HR 0.46; *P* < 0.001)	Progressive disease after ≥1 prior therapy	Not reported	Chan *et al.* (2025)
Axitinib phase 2 (NCT01435122)	Phase 2, single-arm	Extra-pancreatic NET	G1–G2	30	Axitinib (*n* = 30)	PFS	Median PFS 26.7 months (95% CI 11.4–35.1)	Progressive disease after prior therapy	VEGF pathway markers	Strosberg *et al.* (2016)
Pazopanib (NCT00454363)	Phase 2, single-arm	Well-differentiated NETs	G1–G2	52	Pazopanib + octreotide (*n* = 52)	RR	PFS rate at 6 months: PanNET 80.8%, carcinoid 77.8%	Prior SSA treatment	Multiple angiogenic markers	Phan *et al.* (2015)

CgA, chromogranin A; CI, confidence interval; GI-NET, gastrointestinal neuroendocrine tumor; HR, hazard ratio; MKI, multikinase inhibitor; NET, neuroendocrine tumor; ORR, objective response rate; PanNET, pancreatic neuroendocrine tumor; PFS, progression-free survival; RR, response rate; SSA, somatostatin analog; VEGF, vascular endothelial growth factor.

In terms of safety, the CABINET trial reported that 98% of patients in the cabozantinib arm experienced adverse events, with 65% experiencing grade 3 or higher toxicities. The most common grade 3/4 adverse events included hypertension (28%), diarrhea (12%), and fatigue (10%). Dose reductions were required in 65% of patients, and 13% discontinued treatment due to adverse events. Despite these toxicity concerns, the trial demonstrated a favorable risk–benefit profile, given the significant improvement in PFS (Chan *et al.* 2025).

## Unmet needs in the treatment of advanced GEP-NETs

A key question for clinicians in 2024 is which therapeutic option should be chosen after the failure of SSAs, particularly given the expanding array of available treatments. The latest guidelines (i.e., ENETS (Pavel *et al.* 2016) or NCCN) offer multiple treatment options for the same indication in the situation of progressive disease with SSAs (Pavel *et al.* 2016, Shah *et al.* 2018). Moreover, second- and third-line options have only been studied in placebo-controlled trials, lacking head-to-head comparisons for this rare disease. In addition, there is controversy surrounding the optimal treatment sequence due to the absence of controlled groups receiving other sequential treatments. Another challenge is that no clinical parameter or biomarker exists to predict response to a single drug, except in the situation of positive SSTR-2 expression for SSAs and PRRT.

Choosing a systemic targeted treatment as the first-line option for patients with metastasized disease and determining subsequent therapies in the presence of disease progression remains challenging due to the lack of data from head-to-head comparisons of TKIs. This remains an unsolved issue, and current guidelines do not rate or recommend different treatment sequences (Lee *et al.* 2018). Systemic treatment strategies for NET include biotherapy, PRRT, chemotherapy, and targeted therapy. However, there are limited prospective randomized trials directly comparing these modalities.

The NETTER-1 trial was the first prospective randomized trial to evaluate patients with advanced midgut NET and compared PRRT with high-dose somatostatin analog therapy (Strosberg *et al.* 2017). Building on these findings, the NETTER-2 trial (NCT03972488) investigated PRRT (^177^Lu-DOTATATE) plus SSA as the first-line therapy compared to high-dose octreotide LAR in grade 2 and grade 3 GEP-NETs. Recently published results demonstrated that first-line PRRT significantly improved PFS compared to high-dose SSA therapy (median PFS not reached vs 8.5 months; HR 0.28; 95% CI 0.18–0.42; *P* < 0.0001), establishing a new standard of care for treatment-naïve patients with high-proliferative NETs (Strosberg *et al.* 2024). Since these trials, PRRT has been increasingly used earlier in the treatment sequence for GEP-NET patients. However, the optimal positioning within the therapeutic algorithm is still debated and not standardized.

Recent data from the COMPETE trial presented at ENETS 2025, comparing PRRT with everolimus in GEP-NETs, and the OCCLURANDOM trial, evaluating the sequencing of PRRT and systemic therapies, provide important new insights for treatment sequencing. These studies suggest that PRRT may provide superior outcomes compared to TKIs in certain patient populations, although the optimal sequence of PRRT and TKIs remains an area of active investigation. The emerging data indicates that individualized approaches considering tumor characteristics, disease burden, and prior treatments may optimize therapeutic outcomes rather than a one-size-fits-all sequencing strategy.

Ongoing global phase 3 trials, such as the COMPETE study (NCT03049189), comparing everolimus with PRRT (Pavel *et al.* 2018), and the SEQTOR trial (NCT02246127) (Salazar *et al.* 2022) investigating the sequential use of streptozocin-based chemotherapy followed by everolimus or vice versa, aim to provide clarity on treatment sequencing.

Key unmet needs in 2024 include:

- The development of reliable predictive biomarkers for treatment selection and monitoring.
- Understanding optimal sequencing of available therapies, particularly regarding the positioning of PRRT, MKIs, and other systemic treatments.
- Identifying strategies to overcome treatment resistance, particularly to MKIs.
- Establishing effective combination approaches that balance efficacy with tolerability.
- Determining the role of novel MKIs in specific NET subtypes and treatment settings.

In the case of sunitinib and other MKIs, although they have demonstrated effectiveness in increasing PFS and OS in advanced pancreatic NETs, resistance eventually develops in a significant number of patients, posing a challenge in treatment and highlighting the need for further research and alternative strategies (Mateo *et al.* 2012).

Not all patients with advanced GEP-NETs are suitable for surgical treatment. However, some carefully selected patients may still undergo tumor resection at either the primary or metastatic site. This approach is recommended in various clinical guidelines, including those from ESMO, ENETS, and NCCN. For downstaging purposes, a multimodal approach including PRRT, chemotherapy, and targeted therapies such as everolimus and TKIs may be considered, potentially making patients eligible for liver transplantation or other surgical interventions (Shimata *et al.* 2018). Nevertheless, strategies aimed at downstaging the tumor disease pose challenges, and there is a lack of data in this area.

Limited prospective randomized data exist on neo-adjuvant therapies, specifically for NET patients. The Associated Liver Partition and Portal Vein Ligation for Staged Hepatectomy (ALPPS) Registry only documents systemic pretreatment with chemotherapy, while data on TKI or everolimus in this context are completely absent (Linecker *et al.* 2019). Notably, everolimus and most TKIs have shown favorable tumor control rates, including stabilization, partial response, and rarely complete response. These responses typically occur within a few months of starting the medication.

The NETTER-1 trial was the first prospective randomized trial to evaluate patients with advanced midgut NET and compared PRRT with high-dose somatostatin analog therapy (Strosberg *et al.* 2017). Building on these findings, the NETTER-2 trial (NCT03972488) is investigating PRRT (^177^Lu-DOTATATE) plus SSA as the first-line therapy compared to high-dose octreotide LAR in grade 2 and grade 3 GEP-NETs (Singh *et al.* 2024). Preliminary results presented at ENETS 2024 showed that first-line PRRT significantly improved PFS compared to high-dose SSA therapy, potentially establishing a new standard of care for treatment-naïve patients with high-proliferative NETs. Since these trials, PRRT has been increasingly used earlier in the treatment sequence for GEP-NET patients. However, the optimal positioning within the therapeutic algorithm is still debated and not standardized.

## Conclusions

The increasing incidence of NETs worldwide and their often late-stage diagnosis highlights the need for new, effective management strategies. Somatostatin analogs are commonly used as the first-line treatment for symptom control and improved PFS. In the second- and third-line settings for GEP-NETs, options include surgery, PRRT, systemic treatments such as everolimus or sunitinib, and chemotherapy for poorly differentiated tumors. MKIs that inhibit multiple tyrosine kinases have shown promise in treating advanced GEP-NETs, particularly in pancreatic NETs. Pivotal phase 3 clinical studies demonstrated the effectiveness of MKIs in treating advanced pancreatic NETs with longer PFS compared to placebo.

While MKIs offer significant clinical benefits, their toxicity profiles require careful consideration. The high rates of adverse events observed across trials, with 60–98% of patients experiencing some toxicity and 33–77% experiencing grade 3–4 events, emphasize the importance of proactive adverse event management. Common serious toxicities include hypertension, fatigue, diarrhea, and hand-foot syndrome. Despite these challenges, the favorable risk–benefit profile of MKIs supports their use in appropriate clinical settings, provided that adequate toxicity monitoring and management strategies are implemented.

The treatment landscape has evolved significantly with the emergence of new MKIs such as surufatinib, cabozantinib, and lenvatinib, each offering unique targeting profiles and demonstrating promising efficacy in various NET subtypes. The CABINET trial has added cabozantinib as another potential option, while the NETTER-2 trial has provided important insights into the optimal timing of PRRT in the treatment sequence. These developments are reshaping our approach to NET treatment, offering more personalized options for patients.

Nevertheless, the management of advanced GEP-NETs still presents challenges. The lack of clinical parameters or biomarkers impedes personalized treatment approaches. Limited randomized trials comparing different modalities contribute to the complexity. Ongoing studies aim to bring clarity to treatment sequencing, while the resistance to MKIs necessitates further investigation. As research progresses, utilizing current and emerging MKIs, especially in combination with other modalities, hold the potential to significantly advance the treatment landscape for advanced GEP-NETs.

The future directions in NET management will likely focus on developing predictive biomarkers, optimizing treatment sequences, and exploring novel combination strategies. The integration of PRRT earlier in the treatment algorithm, guided by the NETTER-2 results, along with the expanding array of MKIs, provides new opportunities for improving patient outcomes. Continued research into resistance mechanisms and the development of next-generation targeted therapies will be crucial for further advancing the field.

## Declaration of interest

The authors declare that they have NO affiliations with or involvement in any organization or entity with any financial interest in the subject matter or materials discussed in this manuscript.

## Funding

This work was supported by institutional research funding from the University Hospital Zurich and the Hirslanden Klinik Zurich. No external funding was received for this review.

## Author contribution statement

AS conceived the original concept, performed the literature search, and wrote the first draft. JR, GN, and EC contributed to the writing and critical revision of the manuscript. RK, MW, and AP provided critical review and expert input. All authors approved the final version of the manuscript.

## Ethical compliance

All procedures performed in studies involving human participants were in accordance with the ethical standards of the institutional and/or national research committee and with the 1964 Declaration of Helsinki and its later amendments or comparable ethical standards.
